# A portrait of obstructive sleep apnea risk factors in 27,210 middle-aged and older adults in the Canadian Longitudinal Study on Aging

**DOI:** 10.1038/s41598-022-08164-6

**Published:** 2022-03-24

**Authors:** Cynthia Thompson, Julie Legault, Gregory Moullec, Marc Baltzan, Nathan Cross, Thien Thanh Dang-Vu, Marie-Ève Martineau-Dussault, Patrick Hanly, Najib Ayas, Dominique Lorrain, Gillian Einstein, Julie Carrier, Nadia Gosselin

**Affiliations:** 1 Center for Advanced Research in Sleep Medicine, CIUSSS Nord-de-L’Ile-de-Montréal, Montreal, Canada; 2 École de Santé Publique, Département de Médecine Sociale Et Préventive, Université de Montréal, Montreal, Canada; 3 Mount Sinai Hospital Center, Montreal, Canada; 4 Department of Epidemiology and Biostatistics, Faculty of Medicine, McGill University, Montreal, Canada; 5 Institut Universitaire de Gériatrie de Montréal and CRIUGM, CIUSSS du Centre-Sud-de-L’Ile-de-Montréal, Montreal, Canada; 6 PERFORM Centre, Concordia University, Montreal, Canada; 7 Center for Studies in Behavioral Neurobiology, Department of Health, Kinesiology and Applied Physiology, Concordia University, Montreal, Canada; 8 Department of Psychology, Université de Montréal, Montreal, Canada; 9 Sleep Centre, Foothills Medical Centre, Cumming School of Medicine, University of Calgary, Calgary, AB Canada; 10 Department of Medicine, University of British Columbia, Vancouver, BC Canada; 11 Blackmore Sleep Disorders Program, University of British Columbia Hospital, Vancouver, BC Canada; 12 Research Centre On Aging, University Institute of Geriatrics of Sherbrooke, CIUSSS de l’Estrie – CHUS, Sherbrooke, Canada; 13 University of Sherbrooke, Sherbrooke, Canada; 14 Department of Psychology, University of Toronto, Toronto, Canada

**Keywords:** Epidemiology, Population screening, Respiratory signs and symptoms, Comorbidities, Risk factors

## Abstract

Determining the prevalence and characteristics of individuals susceptible to present with obstructive sleep apnea (OSA) is essential for developing targeted and efficient prevention and screening strategies. We included 27,210 participants aged ≥45 years old (50.3% women) from the Canadian Longitudinal Study on Aging. Using the STOP questionnaire combined to the percentage of body fat (%BF), we estimated the prevalence of individuals at high-risk for OSA in a sex and age-specific manner, and tested the relation with comorbidities, menopause and systemic inflammation. The prevalence was 17.5%, and was lower in women (13.1%) than in men (21.9%). A high level of high-sensitivity C-reactive protein was the strongest factor associated with OSA risk and this association was 1.3–2.3 times higher in women than in men. OSA risk increased with age, cardiovascular diseases, diabetes mellitus, anxio-depressive symptoms, asthma and arthritis. In women, post-menopausal status was associated with a high OSA risk. Nearly 1 adult out of 5 older than 45 is at risk for OSA in Canada. Comorbidities, menopause and systemic inflammation, more than age, explain increased OSA prevalence. Considering this high prevalence and associations with medical and mental comorbidities, health care practitioners should incorporate systematic OSA screening in their clinical procedures.

## Introduction

Obstructive sleep apnea (OSA) represents a major public health issue. In fact, nearly one billion adults aged 30–69 years suffer from OSA worldwide^1^. Moreover, as there is a graded increase in OSA prevalence with increasing body mass index (BMI)^2^, it will likely continue increasing with time due to the epidemic of obesity. The association between OSA and medical and mood conditions such as diabetes, hypertension, coronary artery disease, myocardial infarction, congestive heart failure, stroke, and depression^3–5^ is also worrying since a bidirectional relationship is suspected^6–9^. Unfortunately, OSA remains largely under-diagnosed and under-treated^10–13^. The main obstacles are that patients often do not recognize the symptoms, most health professionals do not routinely screen for OSA, and patients find difficult to adhere to continuous positive airway pressure (CPAP) treatment^14^.

Determining the prevalence and characteristics of individuals susceptible to present with OSA in representative population samples is essential for developing more targeted and efficient prevention and systematic screening strategies. This is particularly true for late middle-aged and older adults who are more susceptible to present OSA compared to young adults^15^. Moreover, considering that previous work reported sex differences in how OSA is associated with medical conditions and blood markers (e.g.: apneic women showing higher inflammation levels and being more susceptible to obesity than men)^16,17^, studies now have to include sex differences in their analyses^18^ (Supplementary information).

Investigating OSA prevalence in large cohorts is challenging, as using objective measures of OSA (i.e., polysomnography) is generally limited to smaller samples. To our knowledge, four regional studies used random sampling and polysomnographic recordings to estimate OSA prevalence and assess its association with risk factors and symptoms in adults aged 18–85 in the past 15 years^16,19–21^. Prevalence of mild OSA, defined by an apnea–hypopnea index (AHI) of ≥ 5 but < 15, ranged between 47 and 84% in men, and 25 and 61% in women, while that of moderate-to-severe OSA (AHI ≥ 15) varied between 19 and 50% in men and 8 and 23% in women^16,19,20^. The large variations observed in OSA prevalence were due to its strong positive associations with age and BMI, metabolic comorbidities and menopause^16,19,20^. Other large cohort studies^22,23^ used self-reported diagnosis of OSA obtained through surveys, which probably captures more symptomatic or severe cases, as shown by the drastically lower prevalence that was reported (13–14% in men and 4–6% in women). These results confirm that using self-reported diagnosis, especially in people with mild OSA who are possibly unaware of their condition, leads to an underestimation of OSA prevalence^10,24^.

Another approach to estimate OSA prevalence in large cohorts is using validated screening tools^25–27^. The STOP is based on the Berlin questionnaire and was validated in sleep clinics, surgical and general populations^28,29^. It assesses the self-reported presence of four typical signs and symptoms of OSA, namely **S**noring; daytime **T**iredness; **O**bserved apnea; and high blood **P**ressure, which form the STOP acronym. The STOP-Bang is an alternative scoring algorithm that includes four demographic variables: BMI (B), age (a), neck circumference (n) and sex (g)^28^. Recent systematic reviews reported that a STOP-Bang score of 3 or more has a sensitivity ranging between 88%^30^ and 94%^31^ for detecting moderate-to-severe OSA, but has a much lower specificity (24–42%^30,31^). The STOP / STOP-Bang thus represent useful tools to identify individuals at risk of having a severe form of OSA that should undergo objective polysomnographic testing in clinical settings^31^, as well as to estimate the prevalence of adults susceptible to present OSA (referred as being “at high-risk for OSA” in this article) in large cohorts.

The primary objective of this study was to estimate the sex-specific prevalence of OSA in the national population-based cohort of the Canadian Longitudinal Study on Aging (CLSA) that includes adults aged ≥ 45 years old. We used the STOP questionnaire combined to the percentage of body fat (%BF), an objective measure of obesity that reliably estimates the prevalence of obesity in the CLSA^32^, and accounted for the “B” item of the “Bang”. Sex-specific analyses were stratified according to age, as well as menopausal status for women. A second objective was to identify the sex-specific associations of OSA-related comorbidities and levels of systemic inflammation with high-risk for OSA while controlling for recognized socio-demographic and lifestyle-related confounding variables. Our study represents the largest population-based study (*N* = 27,210 participants) using validated screening questions to estimate sex-specific OSA prevalence, while focusing on adults aged 45–85 who may differ from younger adults in terms of OSA prevalence and determinants.

## Material and methods

### Study design and participants

The CLSA is a national prospective longitudinal study, which includes a total of 51,338 Canadian women and men aged 45–85 years old at the time of recruitment. Participants were separated in two distinct cohorts (comprehensive, *N* = 30,097; tracking, *N* = 21,241) and will be followed until 2033 or death. The CLSA comprehensive cohort recruitment and baseline data collection started in mid-2012 and ended in mid-2015. The design, sampling and source population have been described in detail previously^33^. Our study used the baseline cross-sectional data included in the comprehensive cohort comprising questions pertaining to sleep.

The 30,097 participants of the comprehensive cohort were fluent in English or French, followed in person and living within 25–50 km of one of 11 CLSA data collection sites located in seven Canadian provinces. As evaluated by CLSA’s trained interviewers, participants were free of identifiable signs of cognitive impairment that would prevent them from giving their informed consent and understanding the assessments and tasks. Participants provided information through a computer-assisted interview on aspects relevant to health and aging, and through physical examination, questionnaires, neuropsychological assessment, and biological sample collection during home and site visits. Data pertaining to sleep was collected during home visits.

The CLSA is overseen by a collaborative Research Ethics Board forum chaired at McMaster University, and our own study was approved by the *Centre intégré universitaire de santé et services sociaux du Nord-de-l’Ile-de-Montréal* Research Ethics Board (REB 2018–1584). This research has been performed in accordance with the Declaration of Helsinki. Informed consent was obtained from all participants included in the CLSA.

### Measures

#### Obstructive sleep apnea

We estimated the prevalence of adults susceptible to present OSA using four questions forming the STOP score^28^ combined to the whole body fat percentage with sex-specific cutoffs^34^, resulting in what we refer to as the STOP-Obesity score. For the “B” item, we used the %BF instead of the BMI since the latter underestimates the prevalence of obesity in the CLSA^32^. Neck circumference, one of the 8 questions in the STOP-Bang, is not available in the CLSA cohort, which is why we only used the STOP portion of the questionnaire along with an obesity measure. However, we performed our analyses considering age and sex (“a” and “g” in the acronym, see below).

Snoring was assessed by the yes/no question “Do you snore loudly? By ‘loudly’ I mean louder than talking or loud enough to be heard through closed doors”. Observed apnea was assessed by the yes/no question “Has anyone ever observed you stop breathing in your sleep?”. With respect to high blood pressure, participants were asked the yes/no question “Has a doctor ever told you that you have high blood pressure or hypertension?”. Answers were double-checked with information pertaining to the current intake of hypertensive medication. A score of one was given for each of these questions answered with a “yes”. Daytime tiredness/sleepiness was assessed by the interviewer asking “Over the last month, how often do you find it difficult to stay awake during your normal waking hours when you want to?”, with suggested response categories being “Never”, “Less than once per week”, “Once or twice a week”, “3–5 times per week” or “6–7 times per week”. Participants reporting difficulty staying awake ≥ 3 times per week were considered as experiencing significant daytime sleepiness and were given a score of one. The %BF was measured using a Hologic Discovery A Dual Energy X-Ray Absorptimetry (DXA) machine^35^. Women with a %BF > 35% and men with a %BF > 25% were considered obese, and were given one point for the obesity criterion. The maximum score on the STOP is 4, and participants scoring ≥ 2 are considered at high-risk of having OSA^28^. Our adapted STOP-Obesity score could reach a maximum value of 5 instead of 4, and we considered participants scoring ≥ 3  to be at high-risk for having OSA. We established this cutoff based on recent meta-analyses and studies reporting a high sensitivity for a STOP-Bang score ≥ 3 in patients with mild, moderate and severe OSA^30,31,36,37^. As the CLSA does not include specific questions pertaining to a prior diagnosis of OSA, the terminology “being at high-risk for OSA” refers to the odds of presenting with OSA at the time of testing. It possibly includes individuals who already have OSA and does not exclusively refer to the probability of developing it in the future.

#### Clinical, sociodemographic and lifestyle variables

Clinical, lifestyle and sociodemographic variables related to OSA were selected based on the literature^19,20,23,27,38^, clinical relevance, and availability in the CLSA cohort. Details on the lifestyle and sociodemographic variables are provided in the Supplementary material.

Clinical variables included systemic inflammation, assessed using the level of high-sensitivity C-reactive protein (hs-CRP), and categorized as normal (< 1 mg/L), mild (1–3 mg/L), moderate (3–10 mg/L) and high (> 10 mg/L)^39^. Chronic health conditions diagnosed by a health professional which were expected to, or have already lasted, 6 months or more were considered. This information was reported by the participants, who were asked to answer yes or no to questions about each chronic condition’s diagnosis; all questions systematically started by, “Has a doctor ever told you that you have [chronic medical condition]?”. The chronic medical conditions included were cardiovascular diseases (myocardial infarction, angina pectoris, and/or congestive heart failure), cerebrovascular diseases (stroke and/or transient ischemic attacks), diabetes mellitus, hypothyroidism, arthritis, osteoporosis, memory problems, dementia, anxiety, Parkinson’s disease, chronic obstructive pulmonary disease (COPD) and asthma. The 10-item Center for Epidemiologic Studies Depression scale (CESD-10) was used to assess the presence of depression, where a participant scoring ≥ 10 was considered having depression^40^. Participants who reported having anxiety and/or who scored ≥ 10 on the CESD-10 were considered having an anxious-depressive disorder. Menopausal status was also recorded; women who answered “yes” to “Have you gone through menopause, meaning that your menstrual periods stopped for at least one year and did not restart?” were considered post-menopausal. The others were considered non-menopausal and included pre- and peri-menopausal women.

#### Statistical analyses

Descriptive and regression analyses considered the CLSA inflation and analytic weights, respectively, accounting for the age-stratified sampling strategy and an individual’s probability of inclusion in the CLSA (influenced by unequal sampling probabilities across sampling units and response rates)^41^.

Categorical variables were presented using crude frequencies and weighted proportions. Using chi-square tests, we compared i) the proportion of women and men presenting the individual items of the STOP-Obesity screening tool; ii) the sex-specific estimates of the prevalence of high-risk for OSA between age categories (45–49; 50–54; 55–59; 60–64; 65–69; 70–74; 75–79; ≥ 80); and iii) the estimates of the prevalence of high-risk for OSA between non- and post-menopausal women within age categories. Estimates of the number of middle-aged and older Canadian individuals at high-risk for OSA were calculated using the population of women and men aged ≥ 45 years obtained from the 2016 Canadian Census, multiplied by the sex- and age-specific high-risk for OSA prevalence obtained in the CLSA cohort.

The independent association between high-risk for having OSA and OSA-related comorbidities, level of inflammation, as well as menopausal status for women were tested with multivariate logistic regressions, adjusted for the socio-demographic (age, marital status, ethnicity, income, education level, working status) and lifestyle-related determinants (smoking, alcohol consumption, level of physical activity, sleep duration, sleep quality). Odds ratios > 1 implied that participants presenting a specific comorbidity or a higher-than-normal level of inflammation > 1 mg/L were more likely to be at high-risk for OSA than those who did not present these characteristics.

To better understand whether the sex- and age-specific variations observed in OSA risk can be associated with OSA-related comorbidities and systemic inflammation, we performed sensitivity analyses with the whole cohort (i.e., regardless of OSA risk) using chi-square tests to evaluate: (i) the sex- and age-specific prevalence of comorbidities, and (ii) the sex- and age-specific prevalence of moderate and high levels of systemic inflammation. Moreover, high levels of hs-CRP are known to be associated with obesity^42^, which is a component of our STOP-Obesity score. In order to verify if systemic inflammation was associated with OSA risk independent of obesity, we performed additional sex-specific linear regressions between hs-CRP and each STOP-Obesity item (i.e., snoring; tiredness, sleepiness, fatigue; observed apneas; high blood pressure; obesity) while controlling for all the other items. Results from the sensitivity analyses are presented in the Supplementary material.

Analyses used all participants for whom the variables of interest were available, for women and men, separately. We did not impute missing data. Statistical analyses were performed using IBM SPSS Statistics version 25.0 (Chicago, IL, USA).

### Ethics declaration

The CLSA is overseen by a collaborative Research Ethics Board forum chaired at McMaster University, and our own study was approved by the *Centre intégré universitaire de santé et services sociaux du Nord-de-l’Ile-de-Montréal* Research Ethics Board (REB 2018–1584).

### Consent to participate

Informed consent was obtained from all participants included in the CLSA.

## Results

### Population

**Figure 1:**
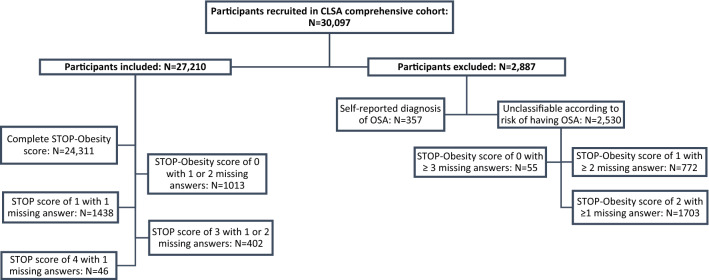
Flowchart describing the inclusion and exclusion of participants for analyses based on the surrogate STOP score.

Of the initial 30,097 participants, we included 27,210 of them (13,799 women and 13,411 men; 59.2 ± 9.8 years old) that we could classify at low- or high-risk for OSA based on their score on the STOP-Obesity (< 3 vs ≥ 3). Of those 27,210 participants, 24,311 answered the four STOP items and had their %BF information available. We classified an additional 2,899 participants although they had one or more missing information on the STOP-Obesity score (Fig. 1). We excluded 2,887 participants, either because they reported having a diagnosis of OSA and no information was available on whether or not they were treated for OSA (*N* = 357), or because they were impossible to classify according to their risk of having OSA (*N* = 2,530). Excluded participants were slightly older, and more likely to be women compared to those included in the analyses.

### Prevalence of adults at high-risk for OSA in the CLSA cohort

Based on a STOP-Obesity score ≥ 3, we classified 17.5% of participants (95%CI: 17.0–18.0%) as being at high-risk for OSA, with a prevalence of 21.9% in men (95%CI: 21.2–22.6%) and 13.1% in women (95%CI: 12.5–13.7%). Table 1 presents sex-specific proportions of participants at low- and high-risk for OSA in each age and menopausal category. Participants at high-risk for OSA were more likely to be 55 years old or more, while women at high-risk for OSA were post-menopausal in a greater proportion than those at low-risk for OSA. Supplementary Table S1 presents detailed socio-demographic, clinical and lifestyle characteristics of women and men at low- and high-risk for OSA. Table 2 presents sex-specific prevalence of each risk factor included in the STOP-Obesity score. Compared to men, women at high-risk for OSA showed a lower prevalence of snoring, observed apneas, and obesity; however, women reported more daytime sleepiness and high blood pressure than men.

**Table 1:** Crude number and weighted proportion of women and men in each age and menopausal category, based to their risk of having OSA (low-risk vs high-risk).

		Women				Men			
		Low-risk		High-risk		Low-risk		High-risk	
		*N*	(%)	*N*	(%)	*N*	(%)	*N*	(%)
Total *N*		11,790	··	2009	··	10,313	··	3098	··
Age (years)	45–49	1286	(16.3)	121	(9.4)	1045	(17.0)	205	(12.0)
	50–54	2048	(26.9)	230	(18.6)	1743	(30.1)	399	(23.9)
	55–59	1965	(14.5)	338	(15.5)	1493	(13.0)	494	(15.4)
	60–64	1974	(15.0)	374	(17.5)	1777	(15.5)	717	(19.0)
	65–69	1593	(10.3)	356	(14.9)	1418	(8.7)	509	(11.1)
	70–74	1078	(6.7)	217	(8.7)	1050	(6.2)	355	(8.0)
	75–79	1138	(6.4)	223	(9.0)	1115	(5.8)	318	(6.9)
	≥ 80	708	(4.0)	150	(6.4)	672	(3.7)	201	(3.8)
Post-menopausal status		7790	(68.7)	1343	(82.3)	··	·· ··		

Low-risk for OSA corresponds to a STOP-Obesity score < 3; High-risk for OSA corresponds to a STOP-Obesity score ≥ 3.

Abbreviations: *N* number of participants, *OSA* obstructive sleep apnea.

**Table 2:** Prevalence (95% CI) of individual STOP-Obesity variables in women and men at low- and high-risk for OSA.

	Women		Men	
	Low-risk (*N* = 11,790)	High-risk (*N* = 2009)	Low-risk (*N* = 10,313)	High-risk (*N* = 3098)
S – Snoring	11.4 (10.2–12.6)	74.7 (73.1–76.3)	21.9 (20.7–23.1)	80.2 (79.0–81.4)
T – Tiredness, sleepiness or fatigue	4.0 (2.9–5.1)	33.8 (31.1–36.5)	4.3 (3.2–5.4)	25.3 (22.8–27.8)
O – Observed apnea	2.8 (1.8–3.8)	46.0 (43.1–48.9)	7.9 (6.9–8.9)	62.7 (59.8–64.6)
P – High blood pressure	20.9 (19.7–22.1)	78.0 (76.8–79.2)	21.2 (20.1–22.3)	71.9 (70.7–73.1)
Obesity – % body fat	68.5 (67.6–69.4)	98.0 (97.7 – 98.3)	59.9 (58.9–60.9)	95.8 (95.4 – 96.2)

### Prevalence by age categories

**Figure 2:**
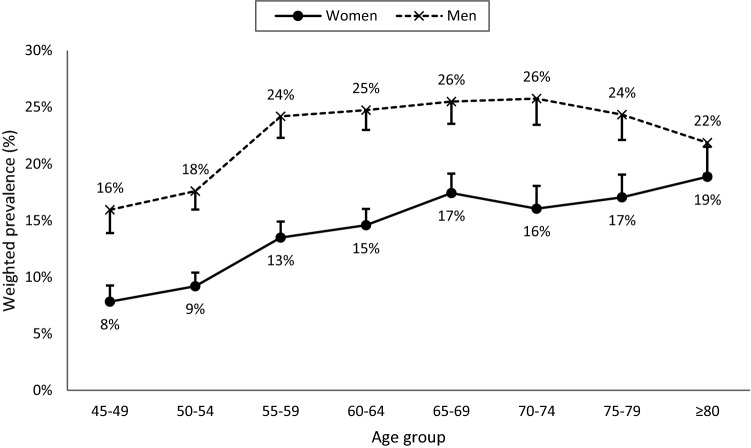
Prevalence of participants at high-risk for OSA by age group in women (solid curve) and men (dashed curve). Error bars represent the 95% confidence interval.

Figure 2 shows the age-specific prevalence of OSA in women and men. High-risk for OSA was 1.2 to 2.0 times more prevalent in men than in women, independent of age. In women, the prevalence of high-risk for OSA increased with age following a quasi-linear trend. In men, this prevalence rather showed an inverted U-shape with a plateau between 55 and 74 years.

### Prevalence by menopausal status

**Figure 3:**
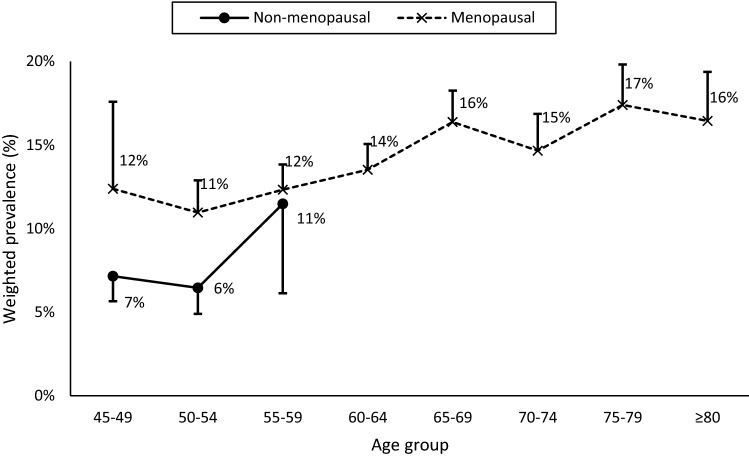
Prevalence of non-menopausal (solid curve) and post-menopausal (dashed curve) women at high-risk for OSA by age. Error bars represent the 95% confidence interval.

The prevalence of high-risk for OSA varied with menopausal status: 7% (95% CI: 6–8%) of non- and 14% (95% CI: 13–15%) of post-menopausal women were at high-risk for OSA. In non-menopausal women, there was an abrupt increase in women aged 55–59 years while in post-menopausal women, OSA risk was relatively stable between 45 and 59 years old, before tending to increase at ages 60 and more (see Fig. 3).

### Extrapolation to the Canadian population

According to the 2016 Canadian Census, approximately five million men and 5.1 million women are aged 45–64 years old in Canada, and 2.9 million men and 3.4 million women are aged ≥ 65. Based on the CLSA cohort average age-specific prevalence of 11.6% in women and 21.4% in men aged 45–64, and 17.8% and 25.0% in older women and men, this corresponds to 1.2 and 1.8 million Canadian women and men, respectively, being at high-risk for OSA.

### Association of clinical and subjective sleep variables with high-risk for OSA

**Figure 4:**
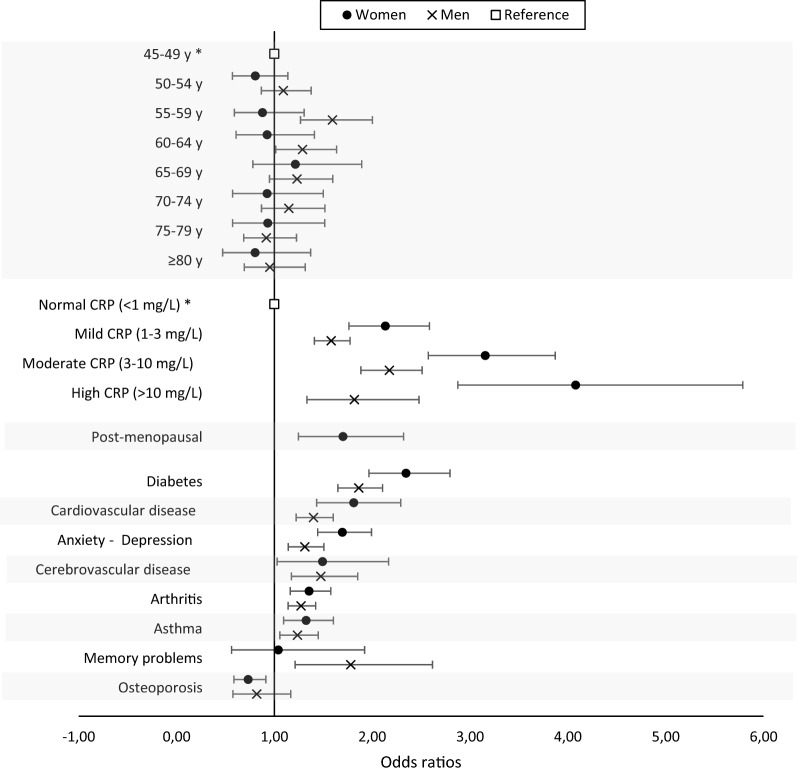
Adjusted odds ratios for high-risk for OSA in women (black circles) and men (grey x-marks) of age, level of systemic inflammation, OSA-related comorbidities and menopausal status with a statistically significant association with risk for OSA. White squares represent the reference categories. Bars represent the 95% confidence intervals. Hypothyroidism, chronic obstructive pulmonary disease, dementia, and Parkinson’s disease were included in the models but are not shown since they were not associated with high-risk for OSA.

Figure 4 illustrates the sex-specific association between individual comorbidities, level of inflammation and menopause (in women) and OSA risk, when adjusted for socio-demographic and lifestyle determinants. Since age is considered as an important risk factor for OSA^15^, we included it in Fig. 4. For clarity purposes, determinants without a significant association with OSA risk (i.e., COPD, hypothyroidism, dementia and Parkinson’s disease) are not shown in Fig. 4 but are presented in the Supplementary Table S2. A level of hs-CRP ≥ 1 mg/L was the strongest independent factor associated with a high OSA risk. The association between systemic inflammation and OSA risk was 1.3 to 2.3 times stronger in women than in men. In fact, in women, the odds ratios increased with higher levels of inflammation, ranging from 2.1 (mild inflammation, hs-CRP between 1 and 3 mg/L) to 4.1 (high inflammation, hs-CRP ≥ 10 mg/L), while in men, they were similar for all hs-CRP levels (1.6, 2.2 and 1.8 for mild, moderate and high inflammation, respectively). Moreover, women and men with diabetes, cardio- and cerebrovascular comorbidities, anxio-depressive symptoms, arthritis and asthma were more likely to be considered at high-risk for OSA. Only in men were memory problems associated with high-risk for OSA, while osteoporosis was associated with lower risk for OSA only in women. Being post-menopausal was also strongly associated with high-risk for OSA.

## Discussion

Our population-based study indicated that 13.1% of Canadian women (or 1.2 million) and 21.9% of Canadian men (or 1.8 million) aged ≥ 45 are at high-risk for presenting OSA and that this risk is 1.2- to twofold higher for middle-aged and older men than women. Cardiovascular diseases, diabetes mellitus, anxio-depressive symptoms, asthma, arthritis and high levels of hs-CRP conferred a greater risk for OSA. The association between systemic inflammation and OSA risk was stronger in women than men. Age alone was not associated with a higher risk for OSA, but comorbidities that typically become more prevalent with aging, as well as menopause in women, showed strong, independent associations with OSA risk. This is the largest population-based study to determine how OSA risk factors vary by sex.

The OSA prevalence estimated in this study is in the range reported in previous non-Canadian studies using screening questionnaires in younger and smaller cohorts (15–31%)^25–27^. However, our estimation is higher than in studies based on self-reported OSA diagnosis (4–14%)^22,23^, supporting the idea that OSA is often undiagnosed. The present estimated OSA prevalence was, however, lower than that observed using polysomnographic recordings in the same age range (19–36% in women, 35–85% in men)^19,20^, suggesting that STOP questions might only capture moderate-to-severe OSA.

Both OSA expression and prevalence are sex-specific: women and men typically report different symptoms, and prevalence is higher in men than in women. We and others found that women with OSA or at high-risk for OSA are less likely to report snoring and witnessed apneas than men^18,43–45^, either because they are embarrassed^45^, or are not notified by a bed partner if they choke or stop breathing. Given that our stereotype of OSA symptoms is based on those reported by men, physicians need to consider women’s atypical symptoms in order to successfully diagnose OSA in women. Our study highlights the need to adapt the concept of “typical” OSA symptoms in order to screen OSA efficiently in women and men. Accordingly, because of the alternative symptoms associated with OSA in women, the sensitivity of OSA screening questions / tools may not be optimal, resulting in an underestimation of the OSA prevalence in women^46^. For example, since the symptoms presented in the STOP-Bang are based on men’s, low scores on the STOP-Bang could, in fact, predict severe forms of OSA in women (sensitivity of 80% in women AHI > 30^47^). Developing and validating OSA screening questionnaires in older women is necessary to ensure a valid epidemiologic portrait of OSA prevalence is large cohort studies.

In women, although the estimated OSA prevalence increases with age, age per se was not independently associated with an increased OSA risk. In the CLSA and other cohorts^19,23,48–51^, menopause was more strongly associated with OSA than age. However, in women aged 55–59 years from our sample, prevalence of OSA risk was similar in non-menopausal and menopausal women. This reflects the continuous nature of the menopausal process, which makes it difficult to classify women in two categories. As such, the lack of difference between the two groups in this age-range might be attributable to the fact that several non-menopausal women were possibly in perimenopause. This lack of difference could also be linked to the prevalence of obesity measured by the %BF and reaching its plateau at age 60, when all women are post-menopausal. Interestingly, studies^48,52,53^ showed that morphological changes in post-menopausal women like increased waist and neck circumferences could account for their increased OSA risk.

Menopause-associated hormonal decreases might also affect OSA risk, where drops in progesterone and 17-ß-estradiol could act on the upper airway dilator muscles and affect ventilation control, airway collapsibility and respiratory drive^23,52,54–56^. Other studies showed that surgical menopause before 50 years old is independently associated with higher OSA prevalence^49^ due to the abrupt post-surgical changes in ovarian hormone levels. OSA risk also tends to be lower in women taking hormone therapy following oophorectomy^49^. A modulating role of ovarian hormones on OSA risk, combined with its effect on morphological changes, is thus possible.

The steep increase in prevalence of high-risk for OSA in men over 55 years is likely related to the concomitant abrupt increased prevalence of diabetes, cardio- and cerebrovascular diseases, arthritis, as well as obesity between 50 and 59 years old. In both women and men, the relationship between obesity and OSA is believed to be bidirectional^2,57^. While obesity is linked to several comorbid conditions aggravated by OSA (e.g., metabolic and cardiovascular diseases) with possible additive effects^16^, OSA could lead to weight gain and obesity by affecting energy metabolism and provoking physical inactivity^2^.

While the risk was similar in men for mild, moderate and high levels of systemic inflammation, increasing levels of inflammation in women conferred a greater OSA risk. Our results build on those reported in a pooled sample of the Nurses’ Health Study, the Nurses’ Health Study II, the Health Professionals Follow-up Study and the Multi-Ethnic Study of Atherosclerosis, in which high CRP levels were associated with increased OSA risk^58^. While inflammation could result from comorbidities and obesity, a recent systematic review and meta-analysis showed increased CRP levels in non-smoking OSA patients without other medical conditions compared to controls^59^. Moreover, results from our sensitivity analyses showed that systemic inflammation was independently associated with STOP-Obesity items other than obesity, especially in women. These results suggest that OSA risk is likely associated with systemic inflammation and that this association is independent of obesity.

Our results confirm that the prevalence of OSA-associated comorbidities is sex- and age-specific^23,46,48,50,60–62^. With the exception of cardiovascular diseases, all comorbidities were more prevalent in women than men at high-risk for OSA, and increased with age in both sexes. Their increased prevalence with age could account for the non-linear association between age and high-risk for OSA, suggesting that the strength of the relationship between OSA risk and comorbidities would become more important than age as individuals acquire years. These results are consistent with our regression models, where age was not independently associated with high-risk for OSA in either women or men aged ≥ 65.

Complex bidirectional, causal relationships may link OSA to several comorbidities^16,63–67^ such as diabetes and cardio- and cerebrovascular diseases^64,68^. Recent bidirectional models proposed that sympathetic excitation associated with cardiovascular diseases^69^, diabetes^63^, and stroke^67^ exacerbates OSA, while OSA-induced hypoxia and sleep fragmentation generate sympathetic activation and inflammation leading to metabolic, cardio- and cerebrovascular diseases^69,70^. Since OSA and chronic medical conditions also share common risk factors such as obesity, age and physical inactivity^3,67^, isolating the independent impact of each comorbidity on the presence of OSA is complicated. The association of each comorbidity with OSA is also likely amplified by the coexistence of different chronic conditions and / or obesity. In fact, the Nagahama study showed the concomitant presence of hypertension, diabetes or metabolic syndrome with obesity additively increased the prevalence of moderate-to-severe OSA^16^.

Considering that most studies controlled for sex without exploring sex differences, our findings that risk factors differentially affect women and men should motivate further investigations of their reciprocity with OSA in both sexes.

The biggest strength of this study is its large sample size, and thus its power allowing the evaluation of sex-specific relationship between several comorbidities and risk factors, and OSA prevalence. The CLSA cohort was sampled to include a proportion of women and men representative of the actual Canadian population, allowing for the generalization of the prevalence of sex-specific risk factors to the Canadian population. It also enabled a precise evaluation of the prevalence of typical and atypical OSA symptoms, especially in women because their symptoms are not often standard and are underrepresented in the OSA literature. Our findings will increase awareness of the high prevalence of individuals at risk for OSA and its different clinical presentation in women, leading to the development of sex-specific prevention and treatment strategies.

A first limitation of this study pertains to the generalizability of our results to participants from other ethnicities, since more than 90% of the participants of our cohort were Caucasian. The CLSA did not over-sample ethnic and aboriginal populations to make the cohort representative of the Canadian population with respect to ethnic/cultural background^33^. Since the underlying pathogenesis for OSA risk could differ by ethnicity, our results might not be representative of non-Caucasian Canadians. Second, another limitation is the cross-sectional analyses which do not allow the determination of causal relationships between risk factors and OSA prevalence. It might also have introduced a non-response bias, since participants unclassified according to OSA risk were older, were more overweight / obese, and more often, women. Third, since neck circumference was not collected in the CLSA cohort, we used the STOP-Obesity to conduct sex-specific analyses instead of the STOP-Bang, which has better predictive values^28,29^. Since neck circumference is a predictor as important as age^71^, prevalence of high-risk for OSA was likely underestimated. Fourth, the CLSA neither asked participants whether they were diagnosed with OSA by a clinician, nor did it collect information about the use of continuous positive airway pressure (CPAP) therapy, which is the most common treatment for OSA. Some participants with OSA might have been misclassified as being at low-risk for OSA after reporting an absence of symptoms assessed by the STOP-Obesity because symptoms were reduced or eliminated by CPAP treatment. Fifth, we estimated prevalence based on the presence of self-reported OSA symptoms and lacked any objective recordings, such as in-laboratory polysomnography or home sleep apnea testing. However, we based our STOP-Obesity cutoff score on recent meta-analyses and studies comparing polysomnography data to STOP-Bang scores ≥ 3^30,31,36,37^. In fact, using this score to detect moderate-to-severe OSA with a cutoff of 3 showed a sensitivity between 88%^30^ and 94%^31^ with a negative predictive value of 93%^30^. Using this same cutoff on the STOP-Obesity (score range: 0 to 5) makes it more severe than on the STOP-Bang (score range: 0 to 8), and has the advantage of reducing the number of false negatives. Sixth, using the STOP-Obesity questionnaire dichotomizes participants as being at low- or high-risk for OSA, which does not account for all possible ranges of OSA.

## Conclusion

This Canadian population-based study offers an updated portrait of middle-aged and older adults at high-risk for OSA. It confirms that being ≥ 55 years old, a male or a post-menopausal woman, overweight or obese, with cardiovascular diseases, diabetes, cerebrovascular disease, anxio-depressive symptoms and higher levels of inflammation are associated with a higher risk for OSA. Given the aging of the population, and increasing prevalence of obesity and chronic health conditions, OSA prevalence will likely continue increasing. An important barrier to OSA diagnosis and treatment is that patients often do not recognize the symptoms, while health professionals do not routinely screen for OSA and sleep issues. It is essential to raise awareness in the general public on the determinants associated with OSA and the impact of OSA for women and men, and to incorporate OSA screening among standard clinical procedures. The CLSA cohort will be followed each 3 years for the next 20 years, allowing a continuing investigation of the temporal relationship between OSA and reported comorbid diseases and symptoms in middle-aged and older adults.

## Supplementary Information

Supplementary Information 1.

Supplementary Information 2.

## References

[1] Benjafield, A. *et al.* Global prevalence of obstructive sleep apnea in adults. *Am. J. Respir. Crit. Care Med.* **American T**, A3962–A3962 (2018).

[2] Carter R, Watenpaugh DE (2008). Obesity and obstructive sleep apnea: or is it OSA and obesity?. Pathophysiology.

[3] McNicholas, W. T., Bonsignore, M. R. & The Management Committee of EU COST ACTION B26. Sleep Apnoea as an Independent Risk for Cardiovascular Disease: Current Evidence, Basic Mechanisms and Research Priorities. *Eur. Respir. J.* **29**, 156–178 (2007) 10.1183/09031936.00027406 17197482

[4] Edwards C, Almeida OP, Ford AH (2020). Obstructive sleep apnea and depression: a systematic review and meta-analysis. Maturitas.

[5] Yeghiazarians Y (2021). Obstructive sleep apnea and cardiovascular disease: a scientific statement from the american heart association. Circulation.

[6] Torres G, Sánchez-de-la-Torre M, Barbé F (2015). Relationship between OSA and hypertension. Chest.

[7] Aurora RN, Crainiceanu C, Gottlieb DJ, Kim JS, Punjabi NM (2018). Obstructive sleep apnea during REM sleep and cardiovascular disease. Am. J. Respir. Crit. Care Med..

[8] Yaggi HK (2005). Obstructive sleep apnea as a risk factor for stroke and death. N. Engl. J. Med..

[9] Uchôa CHG (2015). Impact of OSA on cardiovascular events after coronary artery bypass surgery. Chest.

[10] Braley TJ (2018). Recognition and diagnosis of obstructive sleep apnea in older Americans. J. Am. Geriatr. Soc..

[11] Knauert M, Naik S, Gillespie MB, Kryger M (2015). Clinical consequences and economic costs of untreated obstructive sleep apnea syndrome. World J. Otorhinolaryngol. Neck Surg..

[12] Watson NF (2016). Health care savings: the economic value of diagnostic and therapeutic care for obstructive sleep apnea. J. Clin. Sleep Med..

[13] Laratta CR, Ayas NT, Povitz M, Pendharkar SR (2017). Diagnosis and treatment of obstructive sleep apnea in adults. CMAJ.

[14] Lee CHK, Leow LC, Song PR, Li HH, Ong TH (2017). Acceptance and adherence to continuous positive airway pressure therapy in patients with obstructive sleep apnea (OSA) in a Southeast Asian privately funded healthcare system. Sleep Sci..

[15] Senaratna CV (2017). Prevalence of obstructive sleep apnea in the general population: a systematic review. Sleep Med. Rev..

[16] Matsumoto, T. *et al.* Sleep disordered breathing and metabolic comorbidities across sex and menopausal status in East Asians: the Nagahama Study. *Eur. Respir. J.* **56**, (2020). 10.1183/13993003.02251-2019 32409572

[17] Bouloukaki, I. *et al.* Evaluation of Inflammatory Markers in a Large Sample of Obstructive Sleep Apnea Patients without Comorbidities. *Mediat. Inflamm.* **2017**, (2017). 10.1155/2017/4573756 PMC5555019 28831208

[18] Zhou X (2021). Gender differences of clinical and polysomnographic findings with obstructive sleep apnea syndrome. Sci. Rep..

[19] Heinzer R (2015). Prevalence of sleep-disordered breathing in the general population: the hypnolaus study. Lancet Respir. Med..

[20] Tufik S, Santos-Silva R, Taddei JA, Bittencourt LRA (2010). Obstructive sleep apnea syndrome in the sao paulo epidemiologic sleep study. Sleep Med..

[21] Redline S (2014). Sleep-disordered breathing in Hispanic/Latino individuals of diverse backgrounds: the hispanic community health study/study of latinos. Am. J. Respir. Crit. Care Med..

[22] Appleton SL (2018). Prevalence and comorbidity of sleep conditions in Australian adults: 2016 sleep health foundation national survey. Sleep Heal..

[23] Huang T (2018). Sex differences in the associations of obstructive sleep apnoea with epidemiological factors. Eur. Respir. J..

[24] Young T, Evans L, Finn L, Palta M (1997). Estimation of the clinically diagnosed proportion of sleep apnea syndrome in middle-aged men and women. Sleep.

[25] Hiestand DM, Britz P, Goldman M, Phillips B (2006). Prevalence of symptoms and risk of sleep apnea in the US population. Chest.

[26] Hrubos-Strøm H (2012). Obstructive sleep apnea, verbal memory, and executive function in a community-based high-risk population identified by the berlin questionnaire akershus sleep apnea project. Sleep Breath..

[27] Sunwoo JS (2018). Prevalence, sleep characteristics, and comorbidities in a population at high risk for obstructive sleep apnea: A nationwide questionnaire study in South Korea. PLoS ONE.

[28] Chung F (2008). STOP Questionnaire. Anesthesiology.

[29] Nagappa, M. *et al.* Validation of the stop-bang questionnaire as a screening tool for obstructive sleep apnea among different populations: A systematic review and meta-Analysis. *PLoS One* **10**, (2015). 10.1371/journal.pone.0143697 PMC4678295 26658438

[30] Chen L (2021). Validation of the STOP-Bang questionnaire for screening of obstructive sleep apnea in the general population and commercial drivers: a systematic review and meta-analysis. Sleep Breath..

[31] Pivetta B (2021). Use and performance of the STOP-Bang questionnaire for obstructive sleep apnea screening across geographic regions: A systematic review and meta-analysis. JAMA Netw. Open.

[32] Andreacchi AT (2021). Body mass index, waist circumference, waist-to-hip ratio, and body fat in relation to health care use in the Canadian longitudinal study on aging. Int. J. Obes..

[33] Raina PS (2009). The canadian longitudinal study on aging (CLSA). Can. J. Aging.

[34] Romero-Corral A (2008). Accuracy of body mass index in diagnosing obesity in the adult general population. Int. J. Obes..

[35] Canadian Longitudinal Study on Aging. Bone Mineral Density by Dual- energy X-ray Absorption (DXA) – Whole Body Scan. (2014). Available at: https://clsa-elcv.ca/doc/526. (Accessed: 15 July 2021)

[36] Chiu HY (2017). Diagnostic accuracy of the berlin questionnaire, STOP-BANG, STOP, and epworth sleepiness scale in detecting obstructive sleep apnea: a bivariate meta-analysis. Sleep Med. Rev..

[37] Zheng Z (2021). Comparison of six assessment tools to screen for obstructive sleep apnea in patients with hypertension. Clin. Cardiol..

[38] Kim DH, Kim B, Han K, Kim SW (2021). The relationship between metabolic syndrome and obstructive sleep apnea syndrome: a nationwide population-based study. Sci. Rep..

[39] Pearson TA (2003). Markers of inflammation and cardiovascular disease: application to clinical and public health practice: a statement for healthcare professionals from the centers for disease control and prevention and the American Heart Association. Circulation.

[40] Andresen EM, Malmgren JA, Carter WB, Patrick DL (1994). Screening for depression in well older adults: evaluation of a short form of the CES-D. Am. J. Prev. Med..

[41] Canadian Longitudinal Study on Aging. Sampling and Computation of Response Rates and Sample Weights for the Tracking (Telephone Interview) Participants and Comprehensive Participants. (2017). Available at: https://clsa-elcv.ca/doc/1041. (Accessed: 23 May 2019)

[42] Paepegaey AC (2015). High levels of CRP in morbid obesity: The central role of adipose tissue and lessons for clinical practice before and after bariatric surgery. Surg. Obes. Relat. Dis..

[43] Cairns A, Poulos G, Bogan R (2016). Sex differences in sleep apnea predictors and outcomes from home sleep apnea testing. Nat. Sci. Sleep.

[44] Quintana-Gallego E (2004). Gender differences in obstructive sleep apnea syndrome: a clinical study of 1166 patients. Respir. Med..

[45] Westreich R (2019). The presence of snoring as well as its intensity is underreported by women. J. Clin. Sleep Med..

[46] Bonsignore MR, Saaresranta T, Riha RL, Riha R, Bonsignore M (2019). Sex Differences in obstructive sleep apnoea. Eur. Respir. Rev..

[47] Bauters FA (2020). Sex-specific sleep apnea screening questionnaires: closing the performance gap in women. Sleep Med..

[48] Heinzer R (2018). Impact of sex and menopausal status on the prevalence, clinical presentation, and comorbidities of sleep-disordered breathing. Sleep Med..

[49] Huang T (2018). Type of menopause, age at menopause, and risk of developing obstructive sleep apnea in postmenopausal women. Am. J. Epidemiol..

[50] Mokhlesi B, Ham SA, Gozal D (2016). The effect of sex and age on the comorbidity burden of OSA: An observational analysis from a large nationwide US health claims database. Eur. Respir. J..

[51] Young T, Finn L, Austin D, Peterson A (2003). Menopausal status and sleep-disordered breathing in the Wisconsin Sleep Cohort Study. Am. J. Respir. Crit. Care Med..

[52] Polesel DN (2015). Waist circumference and postmenopause stages as the main associated factors for sleep apnea in women: A cross-sectional population-based study. Menopause.

[53] Davis SR (2012). Understanding weight gain at menopause. Climacteric.

[54] Lozo T, Komnenov D, Badr MS, Mateika JH (2017). Sex differences in sleep disordered breathing in adults. Respir. Physiol. Neurobiol..

[55] Mirer AG (2017). Sleep-disordered breathing and the menopausal transition among participants in the sleep in midlife women study. Menopause.

[56] Jehan, S. *et al.* Sleep Disorders in Postmenopausal Women. *J. sleep Disord. Ther.* **4**, (2015). PMC4621258 26512337

[57] Jehan, S. *et al.* Obstructive sleep apnea and obesity: implications for public health. *Sleep Med. Disord. Int. J.* **1**, (2017). PMC5836788 29517065

[58] Huang T (2021). C-reactive Protein and Risk of OSA in Four US Cohorts. Chest.

[59] Van der Touw T, Smart N, Neil MA (2019). Is C-reactive protein elevated in obstructive sleep apnea? a systematic review and meta-analysis. Biomarkers.

[60] Shepertycky MR, Banno K, Kryger MH (2005). Differences between men and women in the clinical presentation of patients diagnosed with obstructive sleep apnea syndrome. Sleep.

[61] Basoglu OK, Tasbakan MS (2018). Gender differences in clinical and polysomnographic features of obstructive sleep apnea: a clinical study of 2827 patients. Sleep Breath..

[62] Sforza E (2011). Sex differences in obstructive sleep apnoea in an elderly French population. Eur. Respir. J..

[63] Framnes SN, Arble DM (2018). The bidirectional relationship between obstructive sleep apnea and metabolic disease. Front. Endocrinol. (Lausanne).

[64] Meslier N (2003). Impaired glucose-insulin metabolism in males with obstructive sleep apnoea syndrome. Eur. Respir. J..

[65] Somers VK (2008). Sleep apnea and cardiovascular disease. An American Heart Association/American College of Cardiology Foundation Scientific Statement From the American Heart Association Council for High Blood Pressure Research Professional Education Committee. Council on. J. Am. Coll. Cardiol..

[66] Geovanini GR (2018). Association between obstructive sleep apnea and cardiovascular risk factors: variation by age, sex, and race the multi-ethnic study of atherosclerosis. Ann. Am. Thorac. Soc..

[67] Alexiev F (2018). Sleep-disordered breathing and stroke: chicken or egg?. J. Thorac. Dis..

[68] Kapur VK (2017). Clinical practice guideline for diagnostic testing for adult obstructive sleep apnea: an American Academy of Sleep Medicine clinical practice guideline. J. Clin. Sleep Med..

[69] Kasai T, Floras JS, Bradley TD (2012). Sleep apnea and cardiovascular disease: a bidirectional relationship. Circulation.

[70] Shaw JE, Punjabi NM, Wilding JP, Alberti KGMM, Zimmet PZ (2008). Sleep-disordered breathing and type 2 diabetes. A report from the International Diabetes Federation Taskforce on Epidemiology and Prevention. Diabetes Res. Clin. Pract..

[71] Marti-Soler H (2016). The NoSAS score for screening of sleep-disordered breathing: a derivation and validation study. Lancet Respir. Med..

