# Supplementary figures and images for: A portrait of obstructive sleep apnea risk factors in 27,210 middle-aged and older adults in the Canadian Longitudinal Study on Aging
Source: Sci Rep. 2022 Mar 24;12:5127. doi: 10.1038/s41598-022-08164-6 (PMC8948183; doi:10.1038/s41598-022-08164-6)

Supplementary figure S1

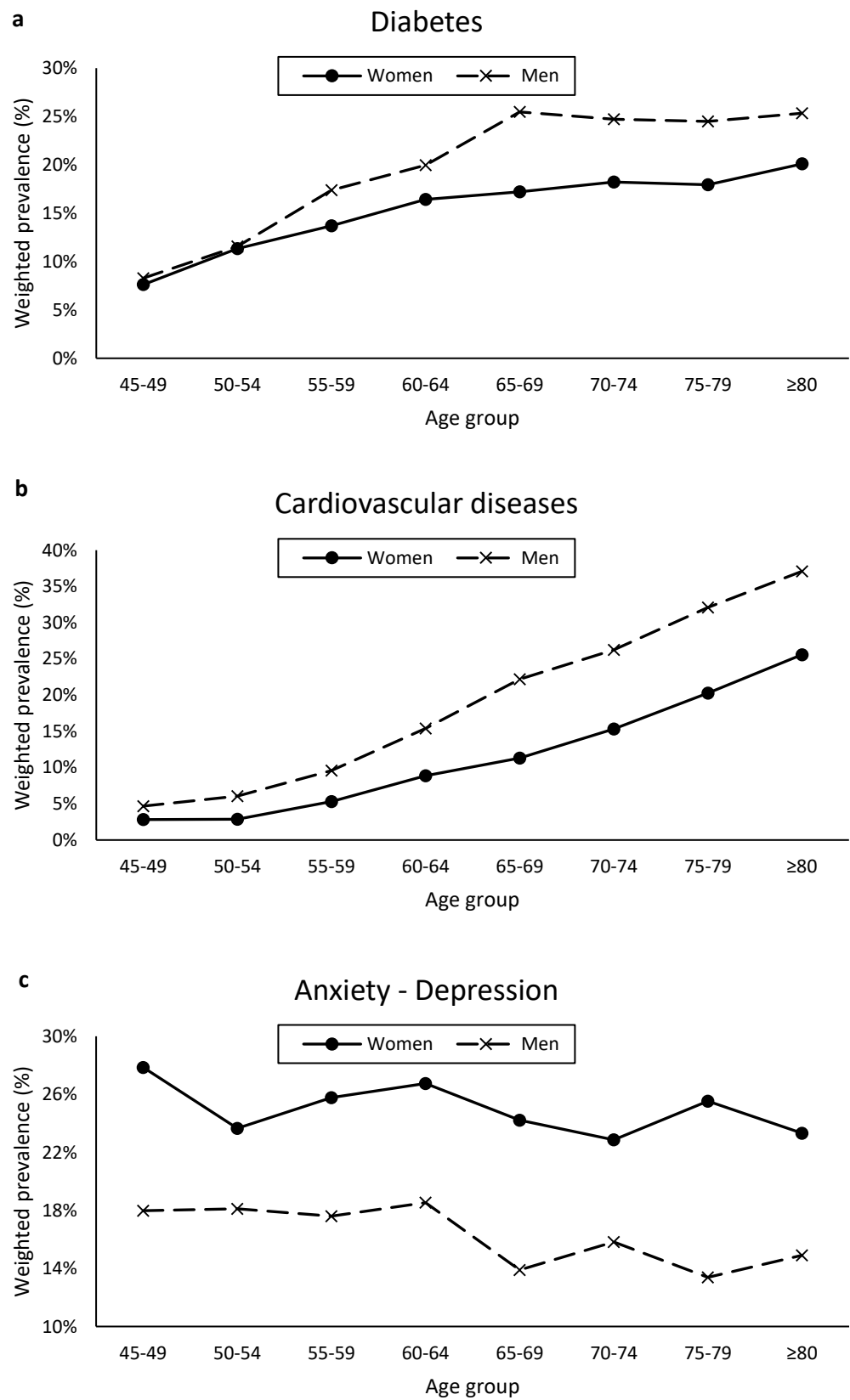

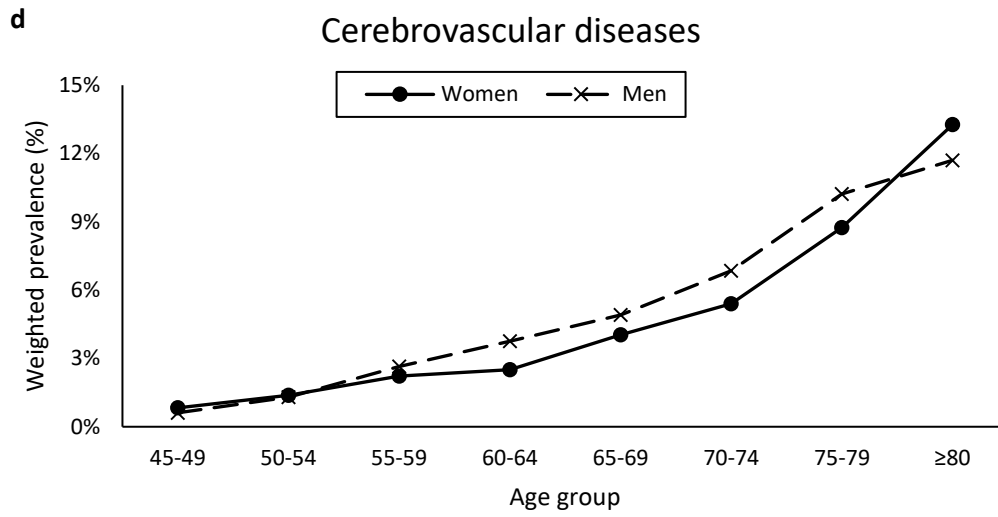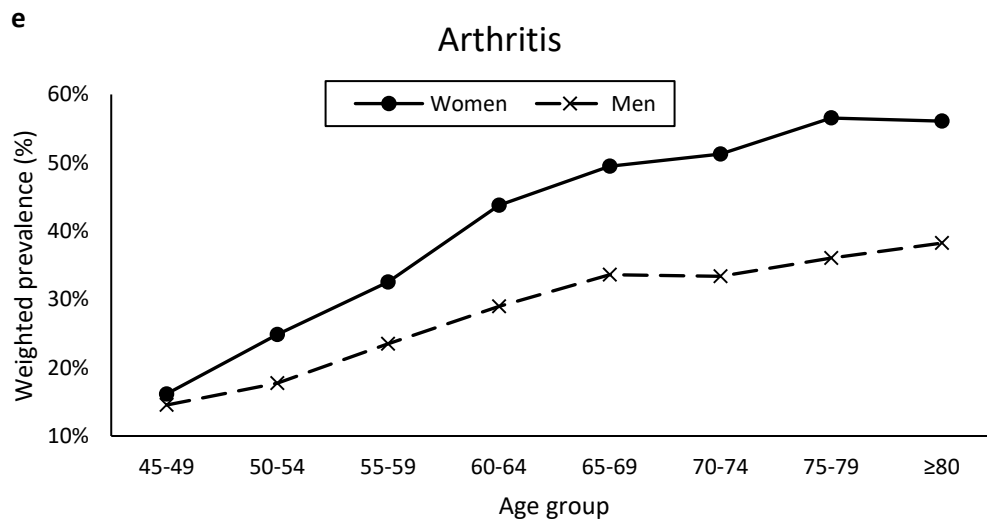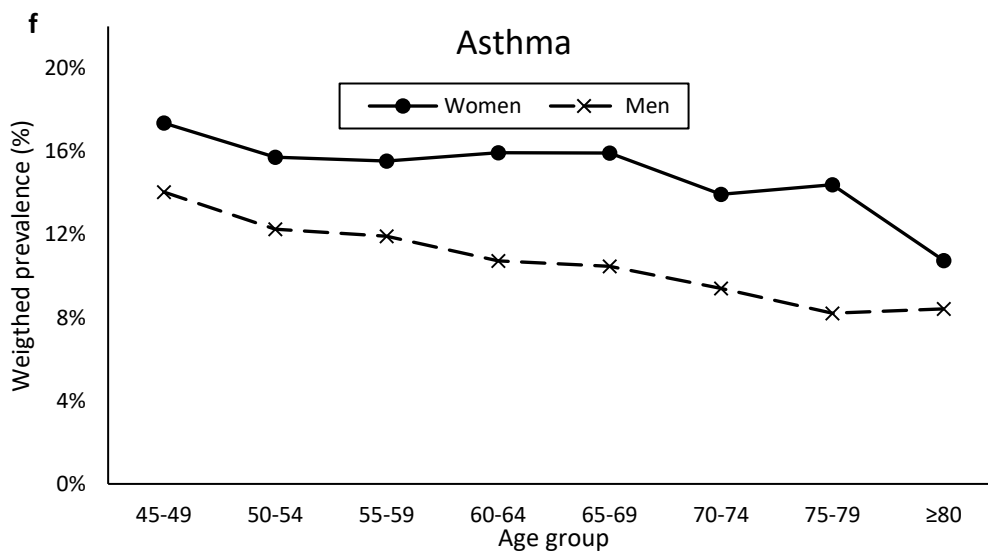

g

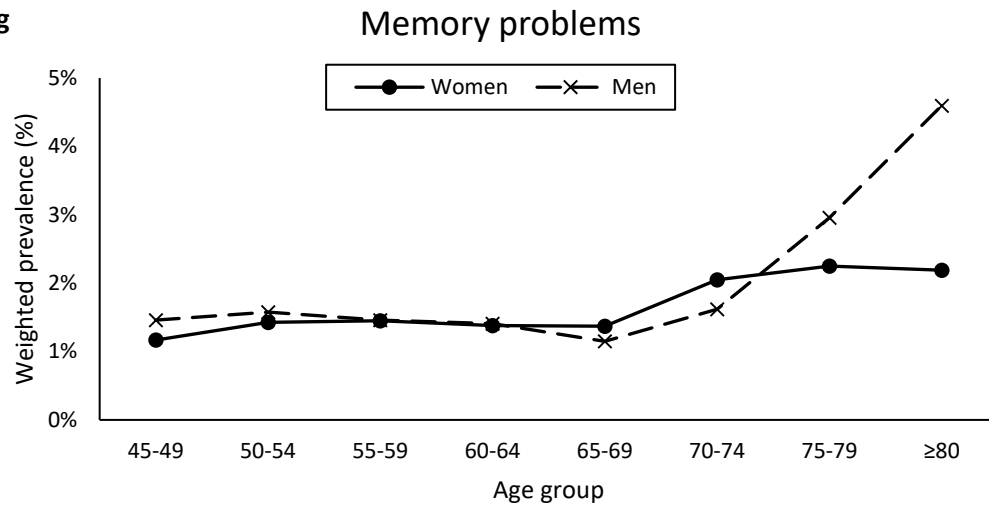

h

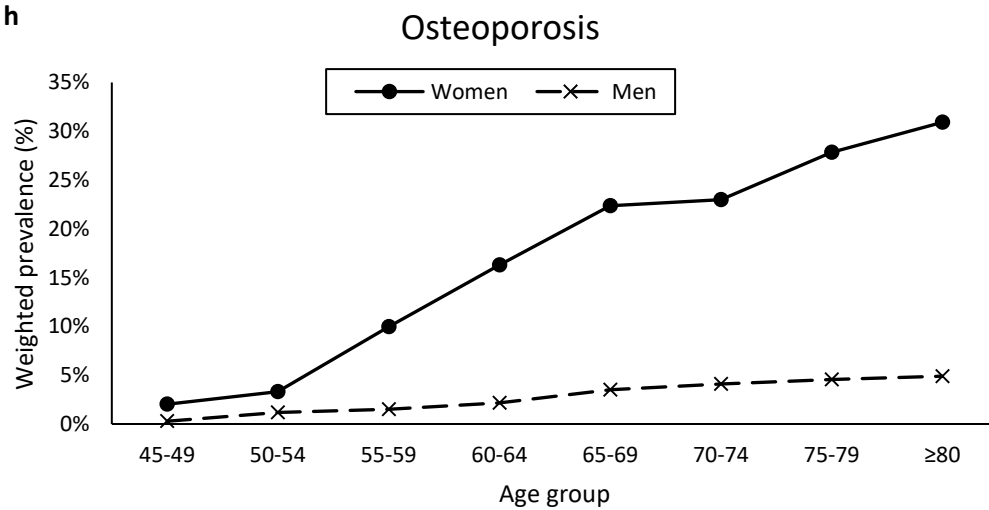

i

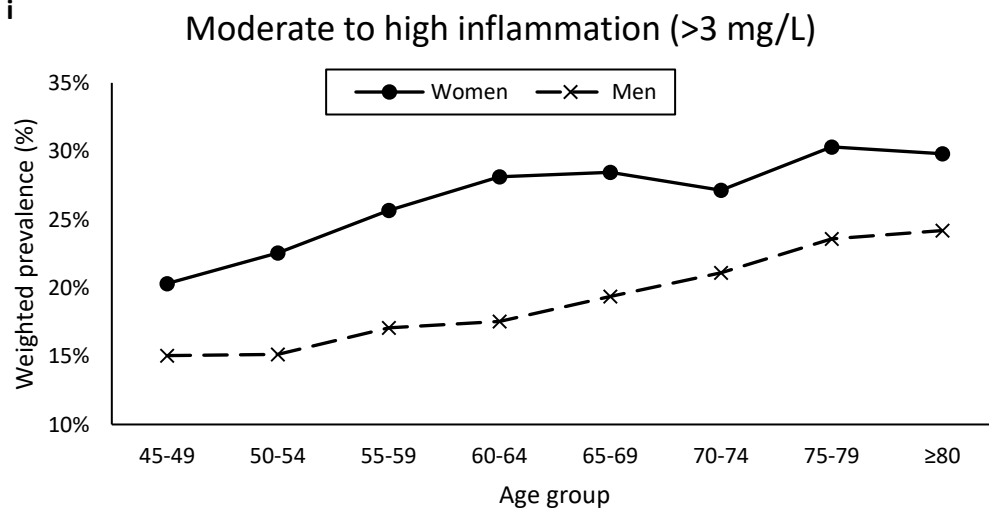

Supplement: Supplementary file 1 — Supplementary Information 1. [file 41598_2022_8164_MOESM1_ESM.pdf]
