# Supplementary material for: A portrait of obstructive sleep apnea risk factors in 27,210 middle-aged and older adults in the Canadian Longitudinal Study on Aging
Source: Sci Rep. 2022 Mar 24;12:5127. doi: 10.1038/s41598-022-08164-6 (PMC8948183; doi:10.1038/s41598-022-08164-6)
Supplement: Supplementary file 2 — Supplementary Information 2. [file 41598_2022_8164_MOESM2_ESM.pdf]

SUPPLEMENTARY – ONLINE RESOURCE 1

A portrait of obstructive sleep apnea risk factors in 27,210 middle-aged and older adults in the Canadian Longitudinal Study on Aging

Cynthia Thompson[1], Julie Legault[1,8], Gregory Moullec[1,3], Marc Baltzan[4,5], Nathan Cross[6,7,8], Thien Thanh Dang-Vu[6,7,8], Marie-Ève Martineau-Dussault[1,2], Patrick Hanly[9], Najib Ayas[10,11], Dominique Lorrain[12,13], Gillian Einstein[14] , Julie Carrier[1,2], Nadia Gosselin[1,2]

[1]Center for Advanced Research in Sleep Medicine, CIUSSS Nord-de-l'Ile-de-Montreal, Montreal, Canada

[2]Department of psychology, Université de Montréal, Montreal, Canada

[3]École de santé publique, Département de Médecine Sociale et Préventive, Université de Montréal, Montreal, Canada

[4]Mount Sinai Hospital Center, Montreal, Canada

[5]Department of Epidemiology and Biostatistics, Faculty of Medicine, McGill University, Montreal, Canada

[6]Institut Universitaire de Geriatrie de Montreal and CRIUGM, CIUSSS du Centre-Sud-de-l'Ile-de-Montreal, Montreal, Canada

[7]PERFORM Centre, Concordia University, Montreal, Canada

[8]Center for Studies in Behavioral Neurobiology, Department of Health, Kinesiology and Applied Physiology, Concordia University, Montreal, Canada

[9]Sleep Centre, Foothills Medical Centre, Cumming School of Medicine, University of Calgary, Calgary, Alberta, Canada

[10]Department of Medicine, University of British Columbia, Vancouver, British Columbia, Canada

[11]Blackmore Sleep Disorders Program, University of British Columbia Hospital, Vancouver, British Columbia, Canada

[12]Research centre on aging, University institute of geriatrics of Sherbrooke, CIUSSS de l'Estrie – CHUS, Sherbrooke, Canada

[13]University of Sherbrooke, Sherbrooke, Canada

[14]Department of Psychology, University of Toronto, Toronto, Canada

Corresponding Author:

Nadia Gosselin, Ph.D.

Center for Advanced Research in Sleep Medicine, Hôpital du Sacré-Cœur de Montréal

5400 Gouin Blvd. West, Office J-5135, Montreal, Quebec, H4J 1C5, Canada

Tel.: +1 514 338 2222, ext. 7717; Fax: +1 514 338 3893; Email: nadia.gosselin@umontreal.ca

SUPPLEMENTARY METHODS

Participants in the Canadian Longitudinal Study on Aging (CLSA) comprehensive cohort were sampled using provincial healthcare registration databases (N = 4,129; 14%) or telephone random digit dialing (N = 25,968; 86%), and divided among seven participating provinces to ensure more precise estimates of various parameters within each national region. The response rate (contact rate * cooperation rate) was 9% for the healthcare registries sampling and 10% for the telephone random digit dialing sampling. Sampling was stratified by sex, age (45-54; 55-64; 65-74 and 75-85) and distance from the data collection sites. The CLSA cohort is representative of the eligible Canadian population and generalizable on many key variables, although the cohort was more educated, had a higher household income, a greater proportion of Canadian-born participants and a better self-reported appreciation of general health[1]. Also, the CLSA comprehensive cohort was recruited from urban regions located 25-50 km from the data collection sites, which limits the generalizability of our findings to populations living in more rural areas.

Sociodemographic and lifestyle-related control variables included age, sex, marital status, ethnicity, income, level of education, working status, smoking, alcohol consumption and level of physical activity, and are detailed in the supplementary material. Marital status was dichotomized as either “living with a spouse” or not, the latter including single, divorced and widowed participants. Ethnicity was dichotomized as “Caucasian” and “Non-Caucasian” based on the self-reported racial / cultural background. Income was stratified as <$20K, $20K-49,9K, $50K-99,9K, $100K-149,9K, >$150K CAD. Four categories were used for education level: less than secondary graduation; secondary graduation without post-secondary education; some post-secondary education; post-secondary education degree / diploma. Information related to working status was stratified as active worker, partly retired, or not working (completely retired / unemployed / housewives).

Lifestyle-related variables pertained to modifiable behaviors and included self-reported smoking, alcohol consumption, level of physical activity, hours of sleep per night and general sleep quality. Three categories were used for smoking status: current smoker, past smoker or never smoker. Alcohol use was stratified based on the long-term health risk determined by the Canadian Guidelines for Low-Risk Drinking[2]: high-

risk, >10 drinks for women and >15 drinks for men weekly; low-risk, 1-10 drinks for women and 1-15 drinks for men weekly; and no drinking. Physical activity was quantified using the Physical Activity Scale for the Elderly (PASE) questionnaire[3] which gives a global score in METS (metabolic equivalent of task) representing the individuals' daily energy expenditure, and quintile cut-off values specific for women and men were used. Self-reported sleep duration and quality duration were also considered. Participants who reported sleeping between 6 and 8 hours per night were considered "normal" sleepers, while those sleeping less than 6 hours or more than 8 hours per night were considered "short" and "long" sleepers, respectively. General sleep quality was assessed by the interviewer asking the multiple-choice question "How satisfied of dissatisfied are you with your current sleep pattern?", with suggested response categories being "Very satisfied", "Satisfied", "Neutral", "Dissatisfied" and "Very dissatisfied". Participants who answered being dissatisfied or very dissatisfied were considered having unsatisfactory sleep quality while the others were considered having satisfactory sleep.

SUPPLEMENTARY RESULTS

Supplementary table S1 shows detailed socio-demographic, clinical and lifestyle characteristics of women and men at low- and high-risk of OSA.

Supplementary table S2 presents the weighted prevalence odds ratios with 95% confidence intervals for high-risk of having OSA in women and men participants for all clinical variables included in the regression models.

Sensitivity analyses of OSA-related medical comorbidities and level of inflammation with respect to age

Because age was not independently associated with high-risk of OSA, we investigated how OSA-related medical comorbidities varied with age in women and men (Supplementary figure S1). Regardless of OSA risk, in both women and men, prevalence of cardiovascular and cerebrovascular comorbidities increased exponentially with age, while the increases in diabetes and arthritis followed a logarithmic trend. Memory problems were more prevalent with increasing age, with an abrupt increase in men aged 70 years old and

more. Osteoporosis was also more prevalent with age, especially in women as compared to men. The prevalence of depression-anxiety disorders decreased at ages 65 and over in both women and men, and the prevalence of asthma also decreased with age. The prevalence of moderate to high systemic inflammation (≥3 mg/L) also increased with age in both women and men. All changes reported from the sensitivity analyses were significant ($p<0.001$).

Sensitivity analyses to verify the association between systemic inflammation and OSA risk, independently of obesity

We conducted sex-specific linear regressions to test the independent association of each STOP-Obesity item with blood hs-CRP level (Supplementary table S3). The results showed that in women, the presence of snoring, tiredness and/or fatigue, observed apneas, high blood pressure and obesity were all significantly associated with higher hs-CRP levels. In men, observed apneas, high blood pressure and obesity were associated with hs-CRP levels, but not snoring nor tiredness.

Supplementary table S1: Crude number and weighted proportion of women and men presenting each socio-demographic, clinical and lifestyle determinant, based to their risk of having OSA (low vs high)

		**Women**				**Men**			
		Low-risk		High-risk		Low-risk		High-risk	
		N	(%)	N	(%)	N	(%)	N	(%)
Total N		11,790	-----	2009	-----	10,313	-----	3098	-----
Marital status	Living with a spouse	7493	73.2	1093	62.6	8127	82.9	2512	84.4
Ethnicity	Caucasian	10,843	91.7	1772	88.2	9376	90.4	2827	90.6
Education	No secondary degree	576	4.5	180	9.0	425	3.5	192	5.3
	Secondary degree	1155	9.5	251	12.7	192	7.4	274	8.4
	No post-secondary degree	832	6.5	202	9.3	698	6.1	250	6.7
	Post-secondary degree	9216	79.5	1373	69.0	8340	83.0	2378	79.6
Working status	Working	5408	56.3	745	44.2	4841	62.7	1258	54.1
	Partly retired	1091	8.6	169	8.8	1305	10.1	447	12.3
	Retired/Unemployed	5232	35.2	1082	47.0	4153	27.2	1390	33.6
Income	<$20,000	614	5.4	122	6.3	512	5.0	168	6.6
	$20-50,000	2486	22.5	453	23.4	2175	22.3	620	21.7
	$50-100,000	3885	35.2	620	32.9	3429	35.7	1041	34.2
	$100-150,000	2136	19.1	358	19.2	1901	19.9	579	20.4
	>$150,000	358	17.7	341	18.2	1638	17.0	459	17.0
Smoking status	No smoker	6176	53.0	905	44.7	4824	50.5	1128	39.5
	Past smoker	4711	38.9	913	45.4	4533	39.7	1166	50.3
	Current smoker	903	8.1	191	9.6	955	9.8	307	10.2
Alcohol	No drinker	2475	21.5	578	31.9	1475	14.4	553	18.2
	Low-risk drinker	6537	68.0	882	58.1	6474	72.8	1733	67.7
	High-risk drinker	1089	10.6	154	10.0	1163	12.8	394	14.2
Sleep duration	Short (<6h)	1503	12.1	441	21.6	1028	10.3	493	16.2
	Normal (6-8h)	9586	81.9	1405	70.3	8812	67.7	2447	79.6
	Long (>8h)	676	6.0	150	8.1	457	3.9	148	4.2
Sleep quality	Satisfactory	8624	73.0	1217	59.5	8273	79.5	2187	69.8
Level of inflammation (hs-CRP)	Normal (<1 mg/L)	4222	42.7	313	18.5	4492	50.3	879	33.4
	Mild (1-3 mg/L)	3842	35.3	683	38.5	3446	35.8	1216	41.5
	Moderate (3-10 mg/L)	2135	19.3	638	35.3	1279	11.7	632	22.1
	High (>10 mg/L)	320	2.7	149	7.8	223	2.2	94	3.0
Comorbidities	Diabetes	1386	10.6	642	30.4	1580	12.9	969	27.2
	Cardiovascular	908	6.4	346	15.6	1491	11.2	728	19.6
	Cerebrovascular	365	2.4	141	6.4	400	2.7	214	5.2
	Anxiety / Depression	2620	22.1	767	39.5	1442	14.4	692	23.1
	Memory problems	152	1.2	49	2.9	136	1.2	81	2.9
	COPD	615	4.3	217	10.7	416	3.3	224	5.8
	Asthma	1670	14.2	456	23.0	1052	10.8	392	13.9
	Arthritis	4474	32.8	1079	51.2	2668	21.9	1095	31.7
	Hypothyroidism	2193	17.7	485	24.3	595	5.2	222	7.1
	Dementia	19	0.2	7	0.3	24	0.1	8	0.3
	Osteoporosis	1764	12.2	297	13.9	241	1.8	77	2.2
	Parkinson's disease	39	0.3	6	0.2	57	0.5	14	0.3
PASE score	Quintile 1	2440	18.0	652	30.3	2319	17.8	904	25.1
	Quintile 2	2542	19.6	476	22.6	2384	19.8	724	20.9
	Quintile 3	2406	20.1	354	18.7	2143	20.3	630	20.8
	Quintile 4	2197	21.0	271	14.3	1803	20.9	462	17.8
	Quintile 5	2126	21.4	232	14.1	1570	21.3	353	15.4

Notes: Low-risk of OSA corresponds to a STOP-Obesity score <3; High-risk of OSA corresponds to a STOP-Obesity score ≥3. Low-risk drinker: 1-10 drinks for women and 1-15 drinks for men, weekly. High-risk drinker: >10 drinks for women and >15 drinks for men, weekly. PASE quintiles use sex-specific cutoff values.

Abbreviations: OSA: Obstructive sleep apnea; COPD: Chronic obstructive pulmonary disease; PASE: Physical activity scale for the elderly; hs-CRP: high-sensitivity C-reactive protein.

Supplementary table S2: Sex-specific weighted prevalence odds ratios with 95% confidence intervals for high-risk of having OSA in relation to age, menopausal status, comorbidities and level of systemic inflammation (hs-CRP)

Factor		Women		Men	
		Beta	OR (95%CI)	Beta	OR (95%CI)
Age	45-49 *	Reference category		Reference category	
	50-54	0.81	0.57 – 1.14	1.09	0.87 – 1.38
	55-59	0.88	0.59 – 1.31	1.59	1.27 – 2.00
	60-64	0.93	0.61 – 1.41	1.29	1.01 – 1.64
	65-69	1.22	0.78 – 1.89	1.23	0.95 – 1.60
	70-74	0.93	0.57 – 1.50	1.15	0.87 – 1.52
	75-79	0.93	0.57 – 1.52	0.92	0.69 – 1.23
	≥80	0.80	0.47 – 1.37	0.96	0.69 – 1.32
Level of inflammation (hs-CRP)	Normal (<1 mg/L) *	Reference category		Reference category	
	Mild (1-3 mg/L)	2.13	1.76 – 2.59	1.58	1.41 – 1.77
	Moderate (3-10 mg/L)	3.16	2.57 – 3.87	2.18	1.88 – 2.51
	High (>10 mg/L)	4.08	2.88 – 5.79	1.82	1.82 – 2.48
Post-menopausal		1.70	1.25 – 2.32	-----	-----
Comorbidities	Diabetes	2.35	1.97 – 2.80	1.86	1.65 – 2.11
	Cardiovascular	1.81	1.43 – 2.29	1.40	1.22 – 1.60
	Anxiety- Depression	1.70	1.44 – 1.99	1.31	1.14 – 1.51
	Cerebrovascular	1.49	1.03 – 2.17	1.48	1.18 – 1.85
	Arthritis	1.35	1.16 – 1.58	1.27	1.14 – 1.42
	Asthma	1.33	1.09 – 1.60	1.24	1.06 – 1.45
	Memory problems	1.04	0.56 – 1.92	1.78	1.21 – 2.62
	Osteoporosis	0.73	0.59 – 0.91	0.82	0.58 – 1.18
	Hypothyroidism	1.17	0.98 – 1.39	1.00	0.81 – 1.23
	Dementia	0.77	0.17 – 3.47	0.59	0.18 – 1.96
	COPD	1.13	0.85 – 1.51	1.09	0.86 – 1.37
	Parkinson’s disease	1.65	0.51 – 5.33	0.69	0.32 – 1.46

Notes: Asterisks indicate the reference categories.

Abbreviations: OSA: obstructive sleep apnea; hs-CRP: high sensitivity C-reactive protein; OR: odds ratio.

Supplementary table S3: Sex-specific, weighted multiple linear regressions testing the association of signs and symptoms used in the STOP-Obesity score to estimate risk of OSA, and blood hs-CRP

		Beta (95% CI)	SE	t	p
Women	Snoring [S]	0.63 (0.42 – 0.85)	0.11	5.78	<0.001
	Tiredness, sleepiness, fatigue [T]	0.54 (0.22 – 0.86)	0.16	3.31	0.001
	Observed apneas [O]	1.08 (0.78 – 1.39)	0.16	6.92	<0.001
	High blood pressure [P]	0.67 (0.48 – 0.86)	0.10	6.97	<0.001
	% body fat >35% [Obesity]	1.50 (1.31 – 1.70)	0.10	15.04	<0.001
Men	Snoring [S]	-0.05 (-0.21 – 0.12)	0.09	-0.54	0.591
	Tiredness, sleepiness, fatigue [T]	0.12 (-0.16 – 0.40)	0.14	0.82	0.410
	Observed apneas [O]	0.32 (0.12 – 0.51)	0.10	3.13	0.002
	High blood pressure [P]	0.48 (0.32 – 0.65)	0.09	5.67	<0.001
	% body fat >25% [Obesity]	0.88 (0.71 – 1.06)	0.09	10.04	<0.001

Abbreviations: OSA: obstructive sleep apnea; hs-CRP: high sensitivity C-reactive protein; CI: confidence interval; SE: standard error.

SUPPLEMENTARY FIGURE CAPTION

Figure S1: Prevalence of A) diabetes; B) cardiovascular diseases; C) anxiety-depression; D) cerebrovascular diseases; E) arthritis; F) asthma; G) memory problems; H) osteoporosis; and I) moderate to high levels of inflammation in women (dark curve) and men (light curve) according to the age group. Data represent prevalence obtained in all women and men, regardless of their risk of having OSA.
